# Uncommon presentation of perforated Meckel’s diverticulum in preterm newborn

**DOI:** 10.1590/0100-3984.2014.0134

**Published:** 2015

**Authors:** Beatriz Regina Alvares, Aline Satomi Yumioka, Gusson Galdino dos Santos

**Affiliations:** 1 Faculdade de Ciências Médicas da Universidade Estadual de Campinas (FCM-Unicamp), Campinas, SP, Brazil.

**Figure 1:**
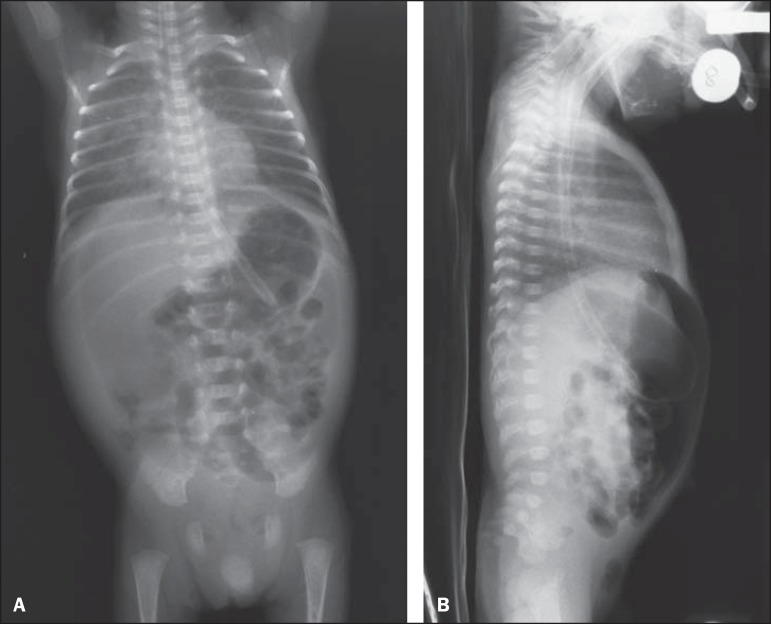
**A:** Chest and abdominal radiography – Image acquired with the patients in supine position, with vertical x-rays, demonstrating hypertransparent abdominal cavity due to accumulation of free air. **B:** Chest and abdominal radiography – Image acquired with the patient in supine position with horizontal x-rays, demonstrating the free air collection located between the anterior abdominal wall and the bowel loops.

*Dear Editor*,

A male neonate with gestational age of 30 weeks, weighting 940 g at birth, with respiratory failure right after birth, and radiological signs compatible with hyaline membrane disease. At his tenth day of life, the patient presented vomiting and abdominal distention, presenting with radiological signs of pneumoperitoneum (Figure 1). 

Initially, the neonate was submitted to peritoneal drainage, due to the lack of surgical conditions, and at the 19th day, after gaining weight and present with hemodynamically stable conditions, was submitted to exploratory laparotomy. During the surgery, a Meckel’s diverticulum (MD) was found, with jejunal perforation, hepatic blockage and obstruction distal to the blockage due to the development of adherence. Resection of about 6 cm of the jejunal loop including the perforated area was performed, with later termino-terminal anastomosis. The anatomopathological result was subacute diverticulitis with ulcer and severe peridiverticulitis. The neonate presented a favorable evolution and was discharged at his 82nd day of life.

Meckel’s diverticulum represents the most common congenital malformation of the digestive tube, and is asymptomatic in most cases^(1-3)^. Symptomatic cases of MD are rarely found, affecting less than 20% of all pediatric cases^(1)^. Bowel obstruction is the most common symptom, usually occurring as a result from inflammation or ileal volvulus^(1,4)^. Meckel’s diverticulum rupture is rarely found in neonates, occurring in less than 10% and manifesting at radiography as pneumoperitoneum^(1)^. In such situations, the differential diagnosis should be made with necrotizing enterocolitis, since this disease is responsible for 41% of cases of neonatal pneumoperitoneum^(4)^.

In the present case, there was a clinical suspicion of necrotizing enterocolitis, but this hypothesis was ruled out as the presence of a perforated MD was intraoperatively confirmed.

The causes of MD include inflammatory reaction, mucosal ulceration and defective muscular layer of the diverticulum^(1,2)^. Rarely, MD perforation may occur as a result from umbilical catheterization by means of an umbilical vein connection with the MD via umbilical cord^(6)^. In the present case, catheterization of umbilical vein and artery was performed with two hours of life; but the late symptoms onset and the exploratory laparotomy demonstrated that the catheterization was not related to the MD perforation.

Hirschsprung’s disease may also predispose to MD perforation due to delayed passage of meconium, determining increased pressure upstream of the diverticulum^(5)^. Such a condition occurs with typical symptoms of bowel obstruction, abdominal pain and bilious vomiting^(5)^. In the present case, despite the symptoms of bowel obstruction and abdominal discomfort at palpation, bilious vomiting was not observed. Furthermore, the histopathological analysis of the surgical specimen ruled out the hypothesis of Hirschsprung’s disease.

Finally, MD should be included as a diagnostic hypothesis in the absence of other factors that might justify the presence of pneumoperitoneum in a neonate. Such a complication is confirmed by means of a surgical procedure.
